# Garlic (*Allium sativum* L.) fertility: transcriptome and proteome analyses provide insight into flower and pollen development

**DOI:** 10.3389/fpls.2015.00271

**Published:** 2015-04-28

**Authors:** Einat Shemesh-Mayer, Tomer Ben-Michael, Neta Rotem, Haim D. Rabinowitch, Adi Doron-Faigenboim, Arkadiusz Kosmala, Dawid Perlikowski, Amir Sherman, Rina Kamenetsky

**Affiliations:** ^1^Agricultural Research Organization, The Volcani Center, Institute of Plant ScienceBet Dagan, Israel; ^2^The Robert H. Smith Faculty of Agriculture, Food, and Environment, The Robert H. Smith Institute of Plant Science and Genetics in Agriculture, The Hebrew University of JerusalemRehovot, Israel; ^3^Department of Environmental Stress Biology, Institute of Plant Genetics of the Polish Academy of SciencesPoznan, Poland

**Keywords:** tapetum, microsporogenesis, gene expression, protein profiling, mitochondrial dysfunction, energy deficiency

## Abstract

Commercial cultivars of garlic, a popular condiment, are sterile, making genetic studies and breeding of this plant challenging. However, recent fertility restoration has enabled advanced physiological and genetic research and hybridization in this important crop. Morphophysiological studies, combined with transcriptome and proteome analyses and quantitative PCR validation, enabled the identification of genes and specific processes involved in gametogenesis in fertile and male-sterile garlic genotypes. Both genotypes exhibit normal meiosis at early stages of anther development, but in the male-sterile plants, tapetal hypertrophy after microspore release leads to pollen degeneration. Transcriptome analysis and global gene-expression profiling showed that >16,000 genes are differentially expressed in the fertile vs. male-sterile developing flowers. Proteome analysis and quantitative comparison of 2D-gel protein maps revealed 36 significantly different protein spots, 9 of which were present only in the male-sterile genotype. Bioinformatic and quantitative PCR validation of 10 candidate genes exhibited significant expression differences between male-sterile and fertile flowers. A comparison of morphophysiological and molecular traits of fertile and male-sterile garlic flowers suggests that respiratory restrictions and/or non-regulated programmed cell death of the tapetum can lead to energy deficiency and consequent pollen abortion. Potential molecular markers for male fertility and sterility in garlic are proposed.

## Introduction

The main functions of the plant reproductive system are flowering, fertilization, and seed development. During flower differentiation, the diploid pollen mother cells undergo meiotic divisions to form tetrads of haploid microspores, which further divide mitotically to vegetative and generative cells of the pollen grain. The latter produce the two gametes required for double fertilization (Borg et al., 2009). Pollen formation, germination and fertilization require high amounts of energy from starch, proteins and sugars (Bhandari, 1984), and anther tissues, mainly the tapetum, play an important supportive role in nourishing the developing microspores (Goldberg et al., 1993). Microsporogenesis is controlled by concerted gene expression in both somatic and gametophytic cells (Mascarenhas, 1990; McCormick, 2004), and some of these genes are anther-specific (McCormick, 2004; Borg et al., 2009). Their function and the effect of their products on pollen development and fertility, however, are obscure, even in model plants.

Garlic (*Allium sativum* L.) represents a sizeable group of useful plants with a large nuclear genome (Arumuganathan and Earle, 1991). The genome of diploid garlic (2n = 2x = 16) is estimated at 15.9 Gbp, 32 times larger than the rice genome. Although full sequencing of the garlic genome remains a challenging task, transcriptome assembly by next generation sequencing may be used for the effective generation of functional genomic data (Kamenetsky et al., 2015).

Classical hybridization, genetic studies, and breeding of this important crop were impossible for hundreds of years, since no commercial garlic varieties produce flowers or seeds. In recent decades, however, seeds have been obtained under experimental conditions from several garlic genotypes (Etoh et al., 1988; Pooler and Simon, 1993, 1994; Jenderek and Hannan, 2000; Kamenetsky et al., 2005). Physiological studies have paved the way for fertility restoration and seed production in bolting genotypes (Kamenetsky et al., 2005; Kamenetsky, 2007; Shemesh et al., 2008), thus facilitating the studies of garlic genetics and breeding (Jenderek and Zewdie, 2005; Rotem et al., 2007, 2011). Bolting and flowering garlic genotypes vary in their reproductive traits, including male fertility and sterility. We have shown that although the temperature has a significant impact on flower development in garlic, in male-sterile plants, fertility cannot be restored by temperature manipulations. We therefore suggested that garlic male sterility is controlled by genetic factors (Shemesh-Mayer et al., 2013, 2015).

In general, the mechanism leading to male sterility in plants is still far from being understood. Male sterility is known to be controlled by genetic and biochemical factors (Hanson, 1991; Mo et al., 1992; Sawhney and Shukla, 1994), or caused by adverse growth conditions, diseases, radiation or chemicals (Islam et al., 2014). Studies in *Arabidopsis*, rice and maize have revealed a large number of genes encoding male fertility, and mutations in any of them could adversely affect the normal development of anthers, or interfere with microgametogenesis, resulting in infertility. Interruptions may occur during chromosome pairing or meiotic segregation, thus resulting in reduced functionality of the tapetum, abnormal pollen-wall formation, inhibition of filament elongation or accelerated anther dehiscence (Scott et al., 2004; Feng and Dickinson, 2007; Chang et al., 2011; Zhang and Yang, 2014). Comparative analyses between the genetic pathways of pollen development in *Arabidopsis*, rice (Wilson and Zhang, 2009), and the bulbous species *Lilium longiflorum* (Mori et al., 2003; Hsu et al., 2007) and *Alstroemeria* (Igawa et al., 2009) have revealed a great similarity in microgametogenesis among plant species, thus indicating a high degree of conservation in the early regulatory network of pollen production.

In plants, three types of inherited male sterility are known: genetic, cytoplasmic, and cytoplasmic–genetic. Genetic male sterility (GMS) is usually regulated by a single recessive nuclear gene with monogenic Mendelian inheritance. The maternally inherited cytoplasmic male sterility (CMS) is under extranuclear genetic control, often associated with unusual open reading frames (ORFs) in the mitochondrial genome (Hanson, 1991; Schnable and Wise, 1998). CMS is known in more than 150 flowering plant species (Laser and Lersten, 1972), which can have N (normal) or aberrant S (sterile) cytoplasm types. In many instances, fertility can be restored specifically by nuclear-encoded fertility-restoring (*Rf*) genes. An interaction between CMS and GMS (cytoplasmic–genetic male sterility, CGMS) is quite common in the genus *Allium* (Budar and Pelletier, 2001). It was identified in bulb onion (*Allium cepa* L.) more than 70 years ago (Jones and Emsweller, 1937; Jones and Clarke, 1943; Havey, 1993, 1995), and later in the bunching onion (*A. fistulosum* L.) (Nishimura and Shibano, 1972; Moue and Uehara, 1985) and in chives (*A. schoenoprasum* L.) (Tatlioglu, 1982). Our living collection of flowering garlic genotypes has enabled us to investigate their reproductive traits and especially fertility/infertility phenomena (Shemesh et al., 2008; Shemesh-Mayer et al., 2013, 2015).

In model plants and in plant species with large and non-sequenced genomes, transcriptome and proteome profiling have revealed marked differences in gene expression during anther and pollen development (Libault et al., 2010; Wei et al., 2010; Kamenetsky et al., 2015). In *Arabidopsis* male meiocytes, the expression of approximately 20,000 genes has been recorded, with more than 800 preferentially expressed in pollen grains. Homologs of these genes have been found in poplar and in rice (Khurana et al., 2012). A comparison between gene-expression profiles of fertile and male-sterile *Arabidopsis* plants has revealed differences between genes associated with the MYB and bHLH protein families (Khurana et al., 2012); in *Brassica napus*, such studies have shown differences related to pollen-wall assembly, binding, catalytic activity, transporter activity, and antioxidant activity (Yan et al., 2013); in chili peppers, differences have been found in ATP synthase, cytochrome oxidase, activated oxygen metabolism and more (Liu et al., 2013).

Proteomic studies have provided information on differential protein profiles between fertile and male-sterile mutants of *Arabidopsis*, tomato, rice, maize and *Brassica* (Imin et al., 2001; Mihr et al., 2001; Wen et al., 2003; Sheoran et al., 2009), and between fertile and sterile garlic genotypes (Shemesh-Mayer et al., 2013). The research presented herein focused on transcriptome and proteome analyses and biological processes occurring during microsporogenesis in fertile and male-sterile garlic genotypes. We identified specific genes and proteins involved in bioenergy balance and hypothesized that an interruption in pollen differentiation is associated with a shortage in bioenergy flow.

## Materials and methods

### Plant material and growth conditions

Following open-pollination, two flowering garlic clones, fertile #87 (F87) and male-sterile #96 (MS96) were raised from two seeds harvested from the same mother plant. The two plants were vegetatively propagated in an insect-proof screenhouse at the ARO, the Volcani Center, Bet Dagan, Israel. In 2012, freshly harvested bulbs were cured and stored in an open shed under ambient conditions, from July to September. After sorting, healthy-looking propagules were stored for 8 weeks at 4°C at a RH of 65–70%. In November, 50 healthy disinfected cloves of each genotype were planted in 20-L containers in a 70:10:20 (v/v) mix of 0.8-mm volcanic tuff particles:perlite:ground coconut peels. Plants were grown under 30% shade in a screenhouse and regularly fertigated with “Shefer” liquid fertilizer (N:P:K = 59:35:94 g L^−1^, Dshanim, Israel).

**Figure 1:**
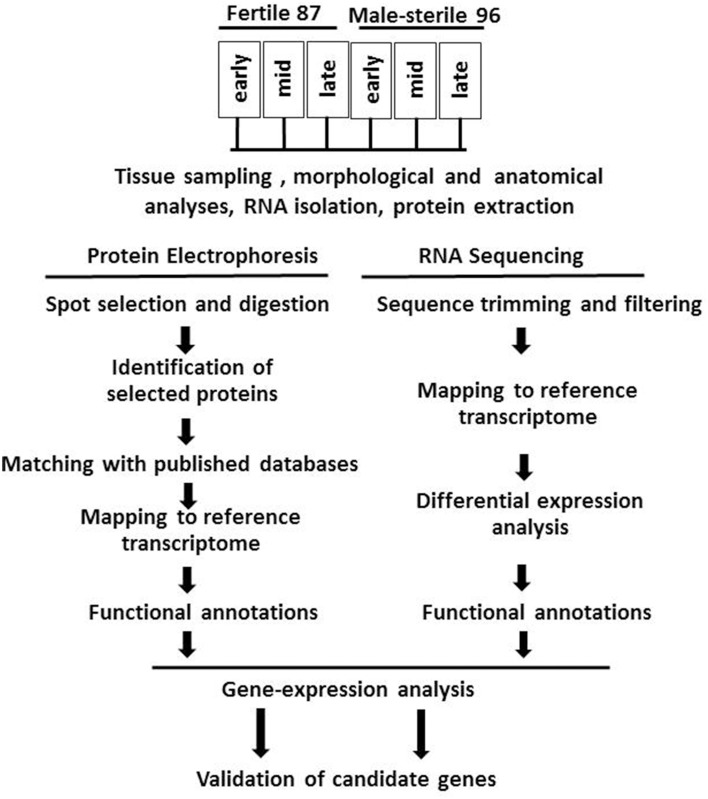
**Flowchart of experimental design, including morphological and anatomical studies of garlic genotypes F87 and MS96, transcriptome and proteome analyses and validation of candidate genes**.

For RNA and proteomic analysis, flowers were collected at the early (green tepal 2.5–3-mm long), mid (green tepal 3–4-mm long) and late (pink tepal 3–4-mm long) stages of development (Shemesh-Mayer et al., 2013). The experimental design is presented in Figure 1.

### Flower phenology, morphology, and anatomy

Flower bud samples were collected in May–June 2013 from spathe break to flower senescence. Morphological studies of 15 flowers per genotype were carried out at each development stage under a stereoscope (Zeiss Stemi 2000-C, Zeiss, Germany). Anatomical studies were performed on flowers fixed in FAA solution (100% acetic acid, 40% formalin, 95% ethanol at 1:2:10 v/v). For plastic embedding, fixed samples were dehydrated in a graded ethanol series and gradually replaced by LR-White resin (Sigma–Aldrich, St. Louis, MO, USA) according to Ruzin (1999). Following polymerization at 60°C for 48–72 h, 2-μm tissue slices obtained by rotary microtome (Leica RM2245) were stained with 0.05% (w/v) toluidine blue and studied under a light microscope (Leica DMLB, Germany).

### Transcriptome analysis

For RNA isolation, tissues were frozen in liquid nitrogen upon sampling and then stored at −80°C until required. Total RNA from flower buds was extracted using RNeasy Mini Kit (Qiagen, Hilden, Germany) according to the manufacturer's instructions. Extract quality was verified using the Agilent 2100 Bioanalyzer with a minimum RNA integrity number value of 7. In total, 18 libraries were prepared, consisting of two genotypes and three replicates of three developmental stages.

RNA library processing was performed by Roy J. Carver Biotechnology Center, W.M. Keck Center for Comparative and Functional Genomics, Urbana, IL, USA. The RNA-Seq libraries were prepared by applying Illumina's “TruSeq Stranded RNA Sample Prep kit” (www.Illumina.com). The pools were quantified by real-time quantitative PCR (rt-qPCR) and sequenced in two lanes each for 101 cycles from one end of the fragments on a HiSeq2000 using TruSeq SBS sequencing kits version 3. Fastq files were generated with Casava 1.8.2. Reads were 100 nt in length.

The sequencing data are deposited in NCBI Sequence Read Archive (SRA) database as bioprojects PRJNA264944 [SSRR1632269-RR1632277] for fertile garlic genotype F87 and PRJNA264945 for male sterile genotype MS96 [SRR1632278-SRR1632288].

Raw reads were cleaned using FASTX Toolkit (http://hannonlab.cshl.edu/fastx_toolkit/index.html, version 0.0.13.2) including:

1. trimming reads' end nucleotides with quality scores <30 using fastq_quality_trimmer,
2. reads consisting of less than 70% base pairs with quality score ≤30 were removed using fastq_quality_filter.

A recently released organ-specific *A. sativum* transcriptome catalog comprising 240,000 contigs from six vegetative and reproductive organs was employed as a reference (NCBI bioproject PRJNA243415, Kamenetsky et al., 2015). The cleaned reads from each library were aligned separately to the transcriptome catalog using Bowtie aligner (Langmead et al., 2009), and the abundance estimation was calculated, via expectation maximization, using the run _RSEM_align_n_estimate.pl script Perl, following the Trinity protocol (Haas et al., 2013).

Differential expression analysis of the sequence count data for each pair of samples (overall seven comparisons) was performed by Bioconductor DESeq package in the R environment (Anders and Huber, 2010). Differential expression was defined as an over four-fold difference in transcription expression with a false discovery-corrected statistical significance of at most 0.001 (i.e., FDR < 0.001).

The transcripts that were differentially expressed in at least one pairwise sample comparison between genotypes and/or developmental stages were examined using cluster analysis. Following the Trinity protocol (Haas et al., 2013), expression normalization was calculated by TMM (trimmed mean of *M*-values) normalization and FPKM (fragments per kilobase of transcript per mllion mapped reads) calculation. Based on the average values in the three replications, hierarchical clustering of transcripts and samples was performed and clusters were extracted using R scripts.

Functional classification of the differentially expressed transcripts was performed using the Blast2GO (Conesa et al., 2005), based on gene ontology (GO) terms obtained from the garlic transcriptome catalog (Kamenetsky et al., 2015). The REVIGO web server (Supek et al., 2011) was used for visualization of the GO terms.

Mitochondrial genes were identified by comparing the garlic transcriptome catalog (NCBI bioproject PRJNA243415) with genomic data for mitochondria of *Arabidopsis thaliana* (NC_001284.2) and *Oryza sativa* Japonica Group (NC_011033.1), using BLASTN algorithm. Further, the 23 sequences that were most similar to the published mitochondrial gene sequences were compared with the NCBI-nr database (http://www.ncbi.nlm.nih.gov).

### Proteome analysis

Upon sampling, fresh flower tissues were dipped in liquid nitrogen, lyophilized and shipped to the Institute of Plant Genetics, Polish Academy of Sciences, Poznan, Poland, where proteins were extracted according to Hurkman and Tanaka (1986). The assay consisted of two biological replicates and three technical repeats. Protein concentration was determined using a 2-D Quant Kit (GE Healthcare, Buckinghamshire, UK), and 2D gel electrophoresis was carried out according to Kosmala et al. (2009) and Perlikowski et al. (2014a). The abundance of each spot was normalized to a relative volume (% volume) and calculated by Image Master 2-D *Platinum* software (GE Healthcare) as the ratio of the volume of each particular spot to the total volume of all spots present on the electrophoretic gel. Liquid chromatography coupled with mass spectrometry was used for identification of the selected proteins in the Laboratory of Mass Spectrometry, Institute of Biochemistry and Biophysics, Polish Academy of Sciences in Warsaw as described in detail by Perlikowski et al. (2014a).

The obtained peptide masses and fragmentation spectra were matched with NCBI-nr using a *Viridiplantae* filter (1,032,142 sequences) and the Mascot search engine (Mascot Daemon v. 2.3.0, Mascot Server v. 2.4.0, MatrixScience). The following search parameters were applied: enzyme specificity was set to trypsin, peptide mass tolerance to ± 30 ppm and fragment mass tolerance to ± 0.1 Da. Protein mass remained unrestricted and monoisotopic mass values were used, but one missed cleavage was allowed. Cysteine alkylation by carbamidomethylation was set as fixed, and methionine oxidation as a variable modification. Proteins were characterized by selection for the highest Mascot-assigned protein score (MudPIT-type) and/or for the highest number of peptide sequences. When only “hypothetical” or “predicted” proteins were identified, amino acid sequence was blasted using the BLASTP algorithm (http://blast.ncbi.nlm.nih.gov/Blast.cgi), and the one with the highest score was selected as its functional homolog (Perlikowski et al., 2014b).

### Comparison between transcriptome and proteome

To compare the transcriptome and proteome data, protein annotations were aligned against the garlic transcriptome catalog (NCBI bioproject PRJNA243415) using TBLASTN algorithm. The matched contigs were compared with the sequences deposited in the NCBI-nr protein database using the BLASTX (Altschul et al., 1990) algorithm with an *E*-value cut-off of 10^−5^. The expression level (in FPKM) of the matched contigs was compared between fertile and male-sterile genotypes.

### Real-time quantitative PCR validation

The expression of 10 selected genes was determined by rt-qPCR using the RNA samples previously employed for the construction of libraries and transcriptome analyses. All rt-qPCR experiments were run in three biological and two technical replications. Garlic homologs of *actin* (AY821677) and *tubulin* (AY148156) served as reference genes for data normalization and calculation of relative amounts of mRNA in the studied samples. The sequences are available in the garlic transcriptome catalog (NCBI bioproject PRJNA243415).

Specific probes were designed for the genes: *nad7, Cox2, 18S rRNA, ccmC, GPAT2*, and *SOD* using Universal Probe Library software (https://www.roche-applied-science.com). The commercial probe numbers, their sequences, primers and efficiency are detailed in Supplementary Table 1. Differential expression was studied using these probes and the reaction mix of FastStart Universal Probe Master (2X; Roche, Indianapolis, IN, USA). The rt-qPCR (Light-Cycler 480 Real-Time PCR System, Roche) analyses were performed with a 20-μl reaction mixture that included 0.5 μM primers, 5 μl cDNA, 1 μl probe and HPLC-grade H_2_O, in a multiwell plate. Each plate had a negative control without cDNA template, and a positive control consisting of plasmid as a template for calibration (Pfaffl et al., 2002). For each primer set, PCR efficiency was determined by the standard curve method. The reactions were performed under the following cycling conditions: an initial denaturing step of 10 min at 95°C, following by 35 cycles consisting of 10 s at 95°C and 30 s at 60°C. The candidate genes were quantified using the ΔΔC_*t*_ method (Pfaffl, 2001), and the *n*-fold change was calculated with LightCycler 480 software, provided by the manufacturer.

The SYBR Green method (Tyler et al., 2004), used to determine the expression of *MS2, MMD1, AP3*, and *Flavonol synthase*, was performed with a 20-μl qPCR reaction mixture consisting of 5 μl template cDNA (1.5 μmol/μl). 10 pmol of each primer and 10 μl of 10X FastStart SYBR Green I Master (Roche). Reactions were performed under the following cycling conditions: an initial denaturing step of 10 min at 95°C, and 35 cycles of 10 s at 95°C, 10 s at 60°C and 10 s at 72°C. The results were analyzed using LightCycler 480 software, provided by the manufacturer.

Both the specific probes and SYBR Green were used for assessment of the reference genes *actin* and *tubulin*.

## Results

### Phenology, morphology, and anatomy of fertile and male-sterile garlic flowers

**Figure 2:**
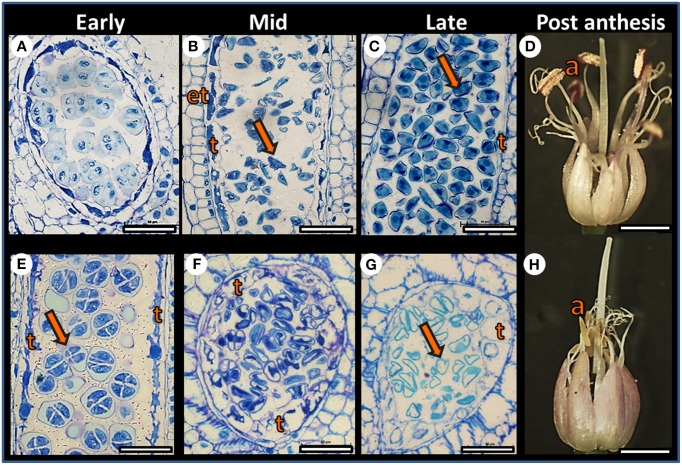
**Comparative developmental anatomy of anthers in the F87 (A–D) and MS96 (E–H) garlic genotypes during microgametogenesis.** Comparisons were made between early, mid and late stages of flower development. **(A)** Cross section of F87 pollen sac at the tetrad stage. Bar = 40 μm. **(B)** Longitudinal section of F87 pollen sac after microspore (arrow) release from the callose. Endothecium (et), and tapetum (t) are visible. Bar = 60 μm. **(C)** Longitudinal section of an anther with mature microspores (arrow) that contain vegetative and generative cells. Tapetum (t) is degenerated, and only remains are visible. Bar = 30 μm. **(D)** Mature F87 flower. Long filaments, dehisced anthers (a) and long style are visible. Bar = 1.5 mm. **(E)** Longitudinal section of MS96 pollen sac at the tetrad (arrow) stage. Typical tapetum (t) cells are visible. Bar = 30 μm. **(F)** Cross section of an MS96 anther, with microspores released from the callose. Note hypertrophy of the tapetum (t) cells. Bar = 45 μm. **(G)** Considerable enlargement of the tapetum (t) cells and degenerated microspores (arrow) in MS96. Bar = 45 μm. **(H)** Mature MS96 flower. Degenerated yellow anthers (a) are visible and the style is elongated. Bar = 1.5 mm.

Plants of both F87 and MS96 genotypes produced dense inflorescences with ca. 300–400 flowers per umbel. However, developmental morphology of the individual flowers varied with genotype. In F87, the filaments of the purple anthers elongated following anthesis; the anthers opened and the pollen dehisced. In contrast, the MS96 anthers turned yellow and degenerated, and no pollen shedding occurred (Figure 2). Female reproductive organs of both genotypes had a similar morphology, appeared to be intact, and when pollinated, both genotypes produced viable seeds.

Anatomical studies at the early stages of anther development revealed normal meiosis in both genotypes (Figures 2A,E); however, differences between genotypes were obvious post-meiosis. Breakdown of the tetrad callose wall of F87 was followed by microspore release into the locular space of the pollen sac, and nourishment was provided by the tapetum layer (Figure 2B). At the late stage of anther development, mitotic divisions and pollen maturation occurred, and the tapetum disintegrated (Figure 2C). In MS96, hypertrophy of the tapetal cells was already observed when the microspores were released (Figure 2F); this was followed by pollen degeneration, including depletion of pollen content and disintegration of the nuclei (Figure 2G).

### Transcriptome analysis of fertile and sterile flowers

Six pools of mRNA samples, representing three floral developmental stages per genotype, served for the construction of high-throughput parallel RNA-Seq libraries. Each of the six cDNA libraries yielded 19–23 million 100-bp one-end reads. Quality trimming and filtration reduced the number to 17–21 million clean reads, ca. 90% of which were mapped to the *de-novo* assembled transcript catalog of *A. sativum* (Table 1).

**Table 1:** **Number of RNA-Seq reads in F87 and MS96 garlic flowers**.

**Genotype**	**Stage of flower development**	**Reads (in millions)**	**Reads discarded after cleaning (%)^a^**	**Clean reads (in millions)^b^**	**Reads mapped to garlic transcriptome catalog (%)^c^**
MS96	Early	22.1 ± 1.2	7.11 ± 0.04	20.5 ± 1.1	89.75 ± 0.29
	Mid	22.7 ± 2.4	7.28 ± 0.10	21.0 ± 2.2	90.42 ± 0.20
	Late	19.2 ± 1.2	7.16 ± 0.05	17.8 ± 1.1	90.33 ± 0.15
F87	Early	20.5 ± 1.4	6.94 ± 0.08	19.1 ± 1.3	89.94 ± 0.79
	Mid	19.9 ± 2.2	7.14 ± 0.08	18.5 ± 2.0	92.66 ± 0.18
	Late	19.2 ± 0.4	7.10 ± 0.06	17.8 ± 0.4	91.84 ± 0.55

Samples, collected in May–June 2013 at early, mid and late stages of flower development, were used for transcriptome analysis. Presented data are means of three replications ± standard error values.

a,b After trimming and filtration.

c Mapping to the transcript catalog of A. sativum (NCBI bioproject PRJNA243415).

**Figure 3:**
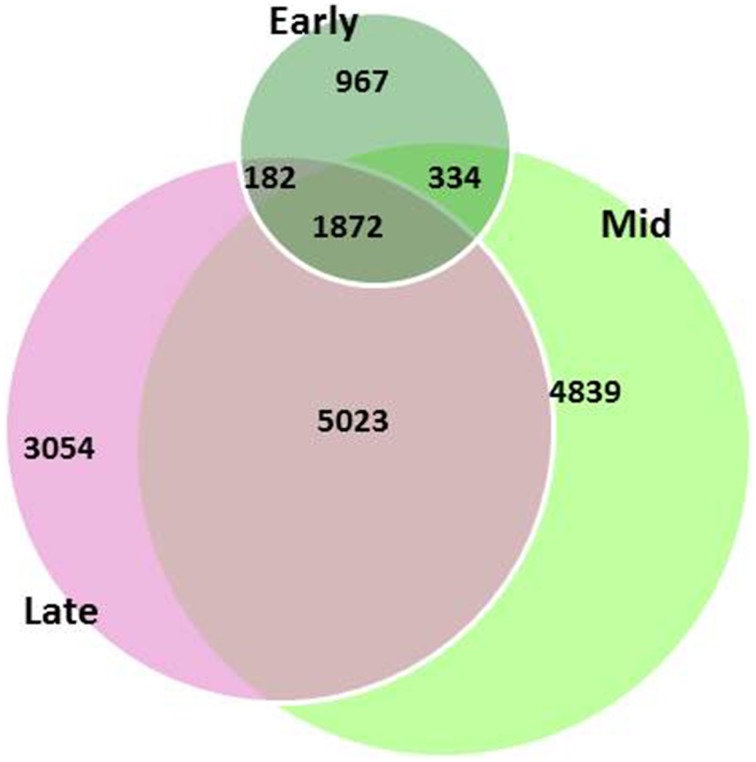
**Differentially expressed genes (DEGs) between garlic genotypes F87 and MS96 at three stages of flower development**. A total of 16,271 DEGs were differentially expressed; 1872 DEGs were common for all three stages, while 967, 4839, 3054 were specific for the early, mid and late stages, respectively.

Global gene-expression profiling showed differential patterns between the two genotypes. A total of 16,271 differentially expressed genes (DEGs) were found between the two genotypes throughout the three developmental stages, but only 12% of the DEGs were common in the flowers throughout all developmental stages; 6% of the DEGs were specific to the early stage, 30% to the mid stage, and 19%—to the late stage of flower development (Figure 3).

**Figure 4:**
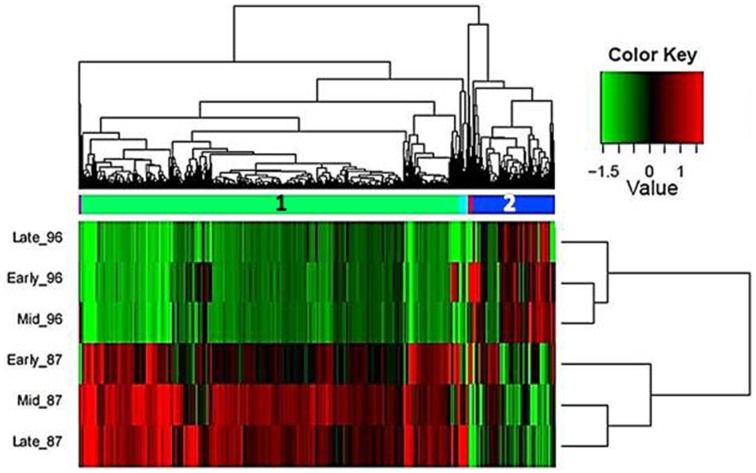
**Hierarchical cluster analysis of gene-expression patterns at the three developmental stages of garlic genotypes F87 and MS96 shows the relative expression levels of each gene (column) in each sample (row).** Two large (1, 2) and three small gene clusters were differentially expressed in one or more samples. The expression values (FPKM; average of three replications) were log_2_-transformed and then median-centered by transcript.

Hierarchical cluster analysis of gene expression revealed clearly differentiated patterns between the two investigated genotypes, with two large and three small gene clusters (Figure 4). Interestingly, in the male-sterile genotype, genes expressed in early and mid stages were clustered together, whereas in the fertile genotype, higher proximity was found between the mid and late stages of flower development, which were distinct from the early stage (Figure 4).

**Figure 5:**
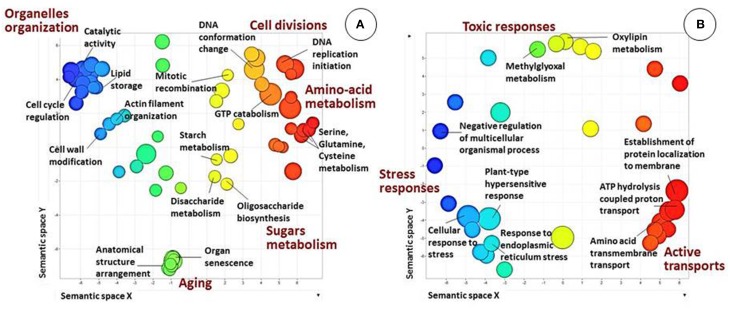
**Biological processes in clusters 1 and 2 (see Figure 4), as revealed by analysis of GO term distribution using Blast2GO and REVIGO algorithms.** GO terms are represented by circles and are plotted according to semantic similarities to other GO terms (adjoining circles are most closely related). Circle size is proportional to the abundance of the GO term in the cluster, while color indicates semantic similarities. Only GO terms with higher than 1% frequency in the cluster are shown. **(A)** Cluster 1. Main patterns are related to the general development of reproductive tissues, metabolism, microsporogenesis and cell-division processes and specific related fertility processes. **(B)** Cluster 2. Main patterns are related to energy-consuming activities and/or response to stress.

Annotation and GO-based functional analysis were performed separately for each of the five DEG clusters. Clusters 1 and 2 had the most substantial differences between the two genotypes (Figure 5).

Bioinformatics analysis revealed that the largest cluster (cluster 1, highlighted in green in Figure 4) consists of 6729 genes, 1194 of which were annotated. At all three developmental stages, most of these genes were highly expressed in genotype F87 compared to genotype MS96. Biological processes, as assessed by Blast2GO, were divided into two main groups (Figure 5A). The first group was related to the general development of reproductive tissues, including, *inter alia*, regulation of meristem development, floral organ development, regulation of cellular-component organization, polysaccharide-catabolism processes, protein polymerization and anthocyanin metabolism. The second group consisted of specific microsporogenesis and cell-division processes, including cell-wall thickening, lipid storage, chromosome organization, DNA replication and packaging, chromatin assembly, mitotic cell cycle, cytoskeleton organization, and dehiscence. Numerous processes specifically associated with fertility (by Blast2GO) were abundant in this cluster, i.e., starch and glycerol-3-phosphate metabolism, hexose biosynthesis, fatty-acyl-CoA transport, naringenin-chalcone synthase activity, Ras and Rab GTPase activity, and more.

Bioinformatics analysis of cluster 2 (highlighted in blue in Figure 4) revealed 1449 genes, 367 of them annotated. In contrast to cluster 1, most of these genes were expressed more strongly in MS96 than in F87. Molecular functions of the annotated genes included the energy-consuming activities of transmembrane transporters, e.g., carboxylic acid and organic acid transmembrane transporters, and ATP synthase (data not shown). Regulation of cellular responses to stress and responses to endoplasmic reticulum stress was abundant in this cluster. Biological processes, assessed by Blast2GO, included, among others, stress responses, energy-coupled proton transmembrane transport, ATP proton transport, and protein targeting and localization to the membrane (Figure 5B).

**Figure 6:**
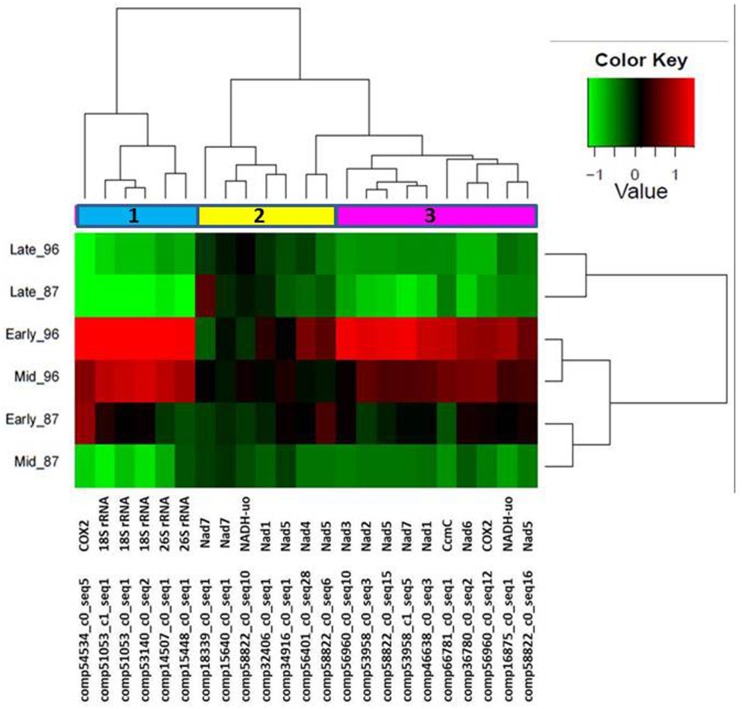
**Hierarchical cluster analysis of the expression patterns of 23 genes with high similarity to the published sequences of plant mitochondrial genes at three flower-development stages of garlic genotypes F87 and MS96.** Three clusters were identified. Note enhanced representation in the early stage of MS96. The relative expression levels of each gene (column) in each sample (row) are shown. Average of three replications' expression values (FPKM) were log_2_-transformed and then median-centered by transcript.

We hypothesized that garlic male sterility might be associated with mitochondrial activity. Therefore, we further selected 23 annotated transcripts with high similarity to the published sequences of mitochondrial genes (Supplemenary Table 2). Hierarchical cluster analysis of the expression patterns of these genes revealed three gene clusters (Figure 6). Most of the identified mitochondrial genes were abundant in the early stage of flower development, and gradually decreased in later stages in MS96 (Figure 6).

### Proteome analysis and transcriptome–proteome matching

For each developmental stage of both genotypes, 2D-gel protein maps were produced. Each map generated ca. 800 protein spots (data not shown). The level of map similarity was very high, but a quantitative comparison revealed 36 protein spots with significant differences, i.e., presence/absence in the genotype or at a specific developmental stage. “Difference” was defined as an at least two-fold change in the normalized volume of a particular spot. The 19 protein spots with the most significant differences in accumulation pattern between genotypes or developmental stages were excised and the proteins identified by mass spectrometry. Out of these, nine proteins were identified in genotype MS96, but not in F87. The predicted protein sequences of these nine proteins were mapped to the garlic transcriptome. Surprisingly, some of these sequences matched a number of transcripts with a high percentage of identity (Table 2).

**Table 2:** **Differentially accumulated proteins and matching transcripts**.

	**Identified protein**	**Accession in NCBI-nr**	**Reference species**	**Matched transcripts in garlic transcriptome catalog**	**Identity (%)**	***E*-value**
1	26S protease regulatory subunit 6A	XP_002271397	*Vitis vinifera*	comp56296_c2_seq6	97	0.0
	homolog A			comp56296_c2_seq7	97	0.0
2	Flavonol synthase	AAO63023	*Allium cepa*	comp47853_c1_seq1	94	0.0
3	ADP-ribosylation factor 1	P51821	*Hyacinthus orientalis*	comp14342_c0_seq1	99	8e-079
				comp14342_c0_seq2	99	8e-079
				comp14638_c0_seq1	98	3e-052
				comp47637_c0_seq1	99	3e-079
				comp47637_c0_seq2	99	3e-079
				comp47637_c0_seq4	99	1e-078
4	Biotin carboxyl carrier protein subunit of Het-ACCase (BCCP2)	XP_002526099	*Ricinus communis*	comp53901_c0_seq11	66	3e-52
5	Putative Cu/Zn superoxide dismutase	BAG16516	*Capsicum chinense*	comp46488_c0_seq1	82	9e-73
6	Cytosolic ascorbate peroxidase	AAY21068	*Capsicum annuum*	comp31384_c0_seq1	85	1e-124
				comp42393_c0_seq1	81	1e-119
				comp58999_c0_seq1	91	1e-084
7	Class-1 LMW heat shock protein	AAM28293	*Ananas comosus*	comp56702_c0_seq11	73	7e-063
				comp56702_c0_seq9	73	7e-063
				comp56702_c0_seq26	73	7e-063
				comp56702_c0_seq20	73	1e-062
				comp56702_c0_seq17	73	1e-062
				comp56702_c0_seq4	73	1e-062
				comp56702_c0_seq19	73	1e-062
				comp56702_c0_seq6	73	1e-062
				comp56702_c0_seq10	73	1e-062
8	Copper/zinc superoxide dismutase, partial	ADB28989	*Allium sativum*	comp46488_c0_seq1	98	2e-084
9	Calmodulin	CAA78058	*Arabidopsis thaliana*	comp39196_c0_seq1	98	3e-074
				comp42519_c0_seq1	91	3e-067
				comp42519_c0_seq3	90	8e-059

Nine proteins were identified only in genotype MS96. The proteins were identified by mass spectrometry analysis, followed by matching with NCBI-nr. The predicted protein sequence was mapped to the garlic transcriptome (NCBI bioproject PRJNA243415) using TBLASTN.

**Figure 7:**
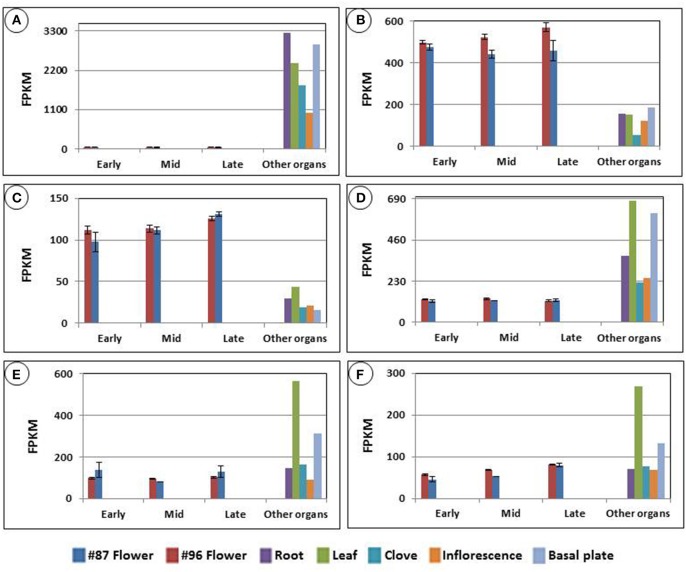
**Six transcripts mapped to the predicted protein ADP-ribosylation factor 1, accumulated in garlic genotype MS96.** The protein was identified by mass spectrometry analysis, followed by matching with NCBI-nr. Expression was calculated using TMM normalization and FPKM calculations. The comparison was made with transcript expression in roots, leaves, cloves, basal plates and inflorescences, using the organ-specific garlic transcriptome catalog (NCBI bioproject PRJNA243415). Transcript-expression levels of **(A)** 14342_c0_seq1, **(B)** 14342_c0_seq2, **(C)** 14638_c0_seq1, **(D)** 47637_c0_seq4, **(E)** 47637_c0_seq1, **(F)** 47637_c0_seq2.

We examined the expression patterns of the transcripts that matched the nine proteins. The previously assembled organ-specific garlic transcript catalog enabled us to compare expression of these transcripts in the developing flowers with those in roots, leaves, cloves, basal plates and inflorescences. For instance, Figure 7 displays the expression of six transcripts mapped to ADP-ribosylation factor 1. Although most transcripts were mapped with high identity to the protein, their expression in garlic tissues differed dramatically. Thus, one of the transcripts was not detected in flowers (Figure 7A), whereas two others were expressed mainly in the reproductive tissues (Figures 7B,C). It should be noted that although at the protein level, the predicted ADP-ribosylation factor 1 was identified exclusively in MS96, transcript accumulation was found in both genotypes, and only one transcript showed significant expression differences between the two genotypes (Figure 7B).

### Data mining for genes involved in male fertility and sterility and validation of candidate genes

Transcriptome- and proteome-assisted mining for the candidate genes associated with microsporogenesis was performed using the following approaches (Supplementary Image 1):

1. Bioinformatics tools were employed to select the number of the DEGs associated with male fertility. The total number of DEGs in fertile vs. male-sterile genotypes was 16,271 (Figure 3). Based on anatomical studies, differences between anthers of the two genotypes were already observed at the mid stage of development (Figure 2), and we therefore focused only on transcripts expressed in the early and mid-stages of development, thus reducing the DEG number to ca. 5500. Furthermore, only annotated DEGs were selected, reducing the list to ca. 1800 DEGs; designating the DEGs expressed in the reference transcriptome catalog only in flowers reduced the number to ca. 350.
2. Revision of the reduced list by GO annotation and manual search for the keywords “male sterility,” “tapetum,” and “pollen” led to selection of the homologs of three genes: *MALE STERILITY 2* (*MS2*) which is associated with pollen development, *MALE MEIOCYTE DEATH 1-LIKE* (*MMD1*) which is associated with anther wall tapetum morphogenesis, and *GLYCEROL-3-PHOSPHATE ACYLTRANSFERASE 2* (*GPAT2*) which is involved in pollen sperm cell differentiation.
3. In addition, a literature search for fertility-related genes led to focusing on homologs of the gene *APETALA 3* (*AP3*) which is known to be associated with morphogenetic processes in the developing anthers and pollen. The sequences of *AP3* were retrieved from NCBI-nr and compared with the garlic transcript catalog using TBLASTN, with an *E*-value cut-off of 10^−5^, and similarity 92% (Table 3).
4. Four annotated genes: *NADH dehydrogenase subunit* 7 (*nad7*), *CYTOCHROME OXIDASE SUBUNIT 2* (*COX2*), *cytochrome C maturation C* (*ccmC*), and *18S rRNA* were retrieved from the list of DEGs with high similarity to the published sequences of the mitochondrial genes. These DEGs were chosen according to their expression pattern at the transcriptome level, mainly if the expression was higher in MS96 in the early and mid-developmental stages.
5. Finally, two proteins from the proteomic data, flavanol synthase and copper/zinc superoxide dismutase (SOD), and their transcripts were chosen from the list of nine proteins that were found only in the MS96 protein analysis. They were chosen according to a literature survey suggesting their involvement in fertility.

**Table 3:** **Transcript sequences of the 10 candidate genes for validation, retrieved from NCBI-nr databases**.

**Transcript number**	**Candidate gene**	**Sequence length (bp)**	**Accession in NCBI-nr**	**Reference species**	**Identity (%)**	***E*-value**
comp35626_c0_seq1	*APETALA 3* (*AP3*)	1062	JX661502.1	*Allium cepa*	92	0.0
comp48389_c0_seq2	*MALE STERILITY 2* (*MS2*)	965	XM_004973057.1	*Setaria italica*	57	1e-101
comp57254_c0_seq52	*MALE MEIOCYTE DEATH 1-LIKE* (*MMD1*)	700	XM_004288545	*Fragaria vesca*	55	6e-013
comp10285_c0_seq1	*GLYCEROL-3-PHOSPHATE ACYLTRANSFERASE 2* (*GPAT2*)	1237	XM_003633174	*Vitis vinifera*	53	5e-081
comp53958_c1_seq5	*NADH dehydrogenase subunit 7* (*nad7*)	1530	DQ381459.1	*Beta vulgaris*	97	0.0
comp66781_c0_seq1	*Cytochrome C* maturation C (*ccmC*)	590	KF798326.1	*Amborella trichopoda*	93	0.0
comp56960_c0_seq12	*CYTOCHROME OXIDASE SUBUNIT 2* (*COX2*)	930	GU253307.1	*Allium cepa*	98	0.0
comp53140_c0_seq2	*18S rRNA*	950	KC465231	*Dendrobium officinale*	99	0.0
comp47853_c1_seq1	Flavanol synthase	1215	AY221247.1	*Allium cepa*	92	0.0
comp46488_c0_seq1	Copper/zinc superoxide dismutase (SOD)	876	GU290312.2	*Allium sativum*	98	0.0

Altogether, 10 candidate genes were retrieved for further validation (Supplementary Image 1, Table 3). Bioinformatics analysis using organ-specific garlic transcript catalog, showed that most of these genes are expressed mainly in flower tissues, and less so in roots, basal plate, leaves and cloves (data not shown). However, two mitochondrial genes (*COX2*, *18S rRNA*) were expressed not only in flowers, but also in the other organs.

**Figure 8:**
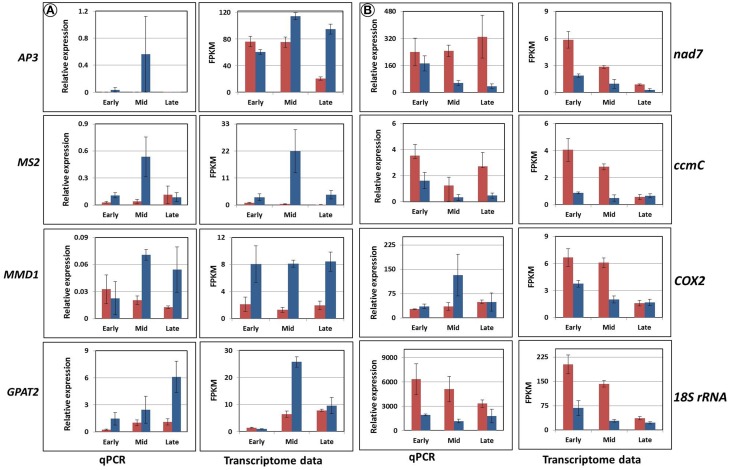
**Validation of the expression of eight candidate genes by rt-qPCR and transcriptome analyses.** Red columns represent gene expression in MS96, blue columns in F87. **(A)** Homologs of the genes *AP3*, *MS2*, *MMD1*, and *GPAT2*, expressed mainly in F87. **(B)** Homologs of the genes *nad7*, *ccmC*, *COX2*, and *18S rRNA*, expressed mainly in MS96.

**Figure 9:**
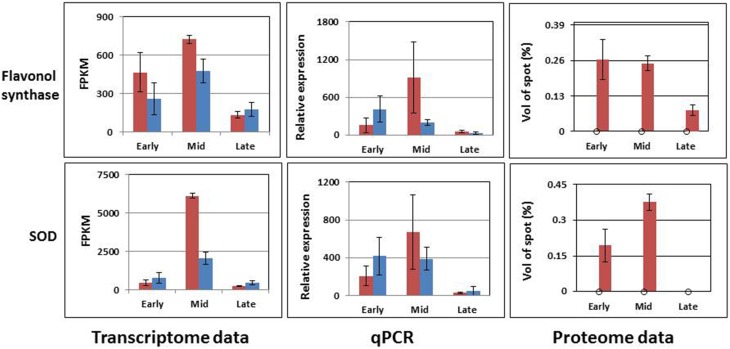
**Accumulation patterns of two predicted proteins and their matched transcripts, as estimated by bioinformatics tools and rt-qPCR.** Red columns represent the expression in MS96, blue columns in F87.

The expression analysis revealed three distinct groups of genes of interest (Figures 8, 9). The first one, abundant in F87, consisted of homologs of *AP3*, *MS2*, *MMD1*, and *GPAT2*, with putative functions in flower and pollen development (Figure 8A). The second group, abundant mainly in MS96, consisted of homologs of the mitochondrial genes *nad7*, *ccmC*, *COX2*, and *18S rRNA* (Figure 8B).

The third group contained two sequences that matched predicted proteins. Although the proteins were detected only in samples from MS96, they were expressed in both genotypes at the transcript level. Moreover, the expression patterns varied between the transcriptome and rt-qPCR data (Figure 9).

## Discussion

The question of fertility and male sterility is of special importance for both genetics and breeding activities in many crops. In garlic, which for years was propagated only vegetatively, this problem is of special scientific interest. The availability of a large collection of flowering garlic genotypes (Kamenetsky et al., 2005; Shemesh et al., 2008) has allowed for in-depth physiological, anatomical, morphological and genetic studies of flowering and seed production. In the present report, we combined the information obtained from morphophysiological studies with analyses of transcriptome and proteome profiles, to identify differences in global gene expression and to pinpoint the specific processes and genes involved in gametogenesis and male sterility in garlic.

### Microsporogenesis and tapetal development in fertile and male-sterile plants

Our histological observations suggested that in young flower buds, microsporogenesis is intact in both fertile and male-sterile genotypes. Later, in the mid and late stages of flower development, differences in pollen differentiation became obvious. Differential expression analysis supported these morphophysiological observations: a relatively low number of genes were differentially expressed during the early stage of anther development, but this number increased significantly in the later stages of development (Figure 3).

Tapetal hypertrophy in the anthers of the male-sterile garlic genotype is associated with pollen deterioration and anther withering prior to first mitosis. In anthers of the fertile plant, no tapetal hypertrophy occurs, pedicels elongate and anthers shed viable pollen (Shemesh-Mayer et al., 2015; Figure 2). Abnormal tapetal development has been reported as one of possible reasons for male sterility in bulb onion (Monosmith, 1928; Saini and Davis, 1969; Holford et al., 1991), *Arabidopsis* (Chaudhury et al., 1994), rice (Nishiyama, 1976), alfalfa (Childers, 1952), and tomato (Rick, 1948), among others. In maize, sunflower and petunia, premature tapetal degeneration is one of the first predictive signs of CMS (Schnable and Wise, 1998). Similarly, in garlic, tapetal hypertrophy, caused by either genetic or abiotic factors, may lead to malnutrition of the released microspores, with the consequent development of empty, non-viable pollen grains (Shemesh-Mayer et al., 2015).

### Gene expression of the fertile and male-sterile genotypes exhibits opposite patterns

In the fertile genotype, vital pollen development is associated with the activity of genes involved in general development of the reproductive tissues, e.g., regulation of meristem structural organization, floral organ development, regulation of cellular-component organization and sugar metabolism (Figure 5). In addition, the processes of cell division associated with microsporogenesis were abundant. Similarly, in *Brassica napus*, maize and petunia, fertility was associated with genes involved in pollen-wall assembly, energy transfer and pollen function and germination (Yan et al., 2013).

Pollen grains are rich in triglycerides, starch and other storage compounds for energy supply during germination, and also contain large amounts of the enzymes involved in glycolysis and mitochondrial respiration (Bhandari, 1984; Dorion et al., 1996; Kerim et al., 2003; Miernyk et al., 2011). In maize, the level of hexoses is much higher in the anthers of male-fertile than male-sterile plants (Datta et al., 2002). Similarly, in fertile garlic flowers, GO analysis has shown an abundance of proteins participating in processes associated with polysaccharides, which are required for energy supply (Figure 5).

Expression analysis of four fertility-related genes (homologs of *AP3*, *MMD1*, *MS2*, and *GPAT2*) confirmed their higher expression in the fertile vs. sterile genotype (Figure 8). The *AP3* gene is a MADS-box transcription factor that controls flower differentiation (Honma and Goto, 2001), and a homolog of this gene might be involved in normal garlic florogenesis. *MMD1*, one of the homologs of *MS1*, is involved in the formation of pollen exine and cytosolic components, as well as tapetum development (Ito et al., 2007), and is also required for male meiosis (Yang et al., 2003). In *Arabidopsis* and rice, mutations in these genes result in lack of normal tapetal programmed cell death (PCD), lack of tapetal DNA fragmentation, delayed tapetal degeneration, abnormal pollen-wall formation and microspore abortion (Ito and Shinozaki, 2002; Li et al., 2011). Similar phenomena were observed in the male-sterile genotype of garlic, with low expression of *MMD1* homolog (Figure 8). We therefore suggest that *MMD1* plays a role in controlling normal pollen formation. *MS2* is strongly related to fertility and encodes a predicted fatty acyl-CoA reductase, involved in fatty-acyl-CoA transport, which is required for exine formation. MS2 protein specifically accumulates in the tapetum. In *Arabidopsis ms2* mutants, microspores collapse shortly after their release from the tetrads, and pollen-wall formation is not detected (Aarts et al., 1997; de Azevedo Souza et al., 2009). In fertile garlic, the *MS2* homolog showed markedly higher expression, mostly at the mid stage of flower development, following microspore release from callose (Figures 2, 8). We therefore propose that this gene might play an essential role in pollen formation, and can be considered a potential marker for fertility in garlic. Members of the *GPAT* family are involved in pollen development and tapetum viability in *Arabidopsis* (Zheng et al., 2003). Similarly, abundance of the *GPAT2* homolog in the fertile genotype indicates its involvement in normal functioning of the garlic tapetum (Figure 8).

### Male-sterility in garlic is associated with respiration restriction (the energy deficiency model)

According to the GO analysis, the flowers of male sterile garlic have a higher abundance of genes associated with energy flow, respiration and mitochondrial functions. In general, in higher plants, CMS is associated with alterations in the mitochondrial genome that affect the functions of anthers, pollen or male gametes. The genes that determine CMS have chimeric open-reading-frames (ORFs) of unknown origin and function (Woodson and Chory, 2008; Shaya et al., 2012; Islam et al., 2014). Many of these genes consist of portions of essential mitochondrial genes involved in respiration pathways, including *NADH-dehydrogenase subunit* genes, *cytochrome oxidase*, and *ATP synthase*. Such fused genes may disrupt the mitochondrial function by impairing its membrane integrity and interfering with the expression of intact mitochondrial genes, with a consequent reduction in respiration and energy supply (Chen and Liu, 2014). This phenomenon has been described as the “Energy Deficiency Model” (Woodson and Chory, 2008; Chen and Liu, 2014). In male-sterile garlic, a relatively high expression of mitochondrial genes is already noted at the meiotic stage, prior to the appearance of the anatomical evidence of sterility (Figure 6). Later, when tapetum heterotrophy and pollen abortion are evident in the male-sterile anthers (Figure 2, Shemesh-Mayer et al., 2015), mitochondrial gene expression decreases (Figure 6). We therefore propose that in garlic, similar to other plant species (Woodson and Chory, 2008; Shaya et al., 2012; Chen and Liu, 2014), the chimeric ORFs might be fused to mitochondrial genes, impairing their respiratory system. Furthermore, the internal feedback from low energy in the cell urges the mitochondria to overcome the shortage, thus resulting in a high expression of mitochondrial genes.

This hypothesis is supported by higher expression of three specific mitochondrial genes in male-sterile as compared to fertile flowers (Figure 8). The association between these genes and CMS has been also reported in sterile phenotypes of *Petunia* and *Nicotiana sylvestris* (*NADH-dehydrogenase subunit* gene; Rasmussen and Hanson, 1989; Pla et al., 1995), in *Brassica napus* (*ccmC*, a participant in the respiratory complex; Leino et al., 2005; Kim et al., 2009b) and in maize (*18S rRNA*; Wen and Chase, 1999). Although *COX2* is associated with CMS in sugar beet (Senda et al., 1991), our analysis of the garlic *COX2* homolog gave controversial results in the transcriptome analysis vs. qPCR validation, probably due to low sensitivity of the primers.

### Oxidative stress might induce male sterility in garlic (the aberrant PCD model)

The “Aberrant PCD Model” suggests that the increased production of reactive oxygen species (ROS) in mitochondria stimulates unregulated PCD in the tapetum, resulting in pollen abortion (Chen and Liu, 2014; Touzet and Meyer, 2014). Mitochondrial ROS, generated by oxidative stress, are abundant in the anthers of the CMS lines of sunflower, cotton and rice (Jiang et al., 2007; Touzet and Meyer, 2014). In male-sterile garlic, this model might explain the results of the GO analysis, which indicated processes of oxidative stress and negative regulation of PCD, suggesting premature tapetal PCD and consequent microspore degeneration (Figure 5B).

### Correlation of mRNA and protein accumulation

Numerous reports have suggested that RNA transcript accumulation is not always conveyed to the final product—protein. For example, a negative correlation between mRNA and protein accumulation patterns was found in *Arabidopsis* in response to cold treatment (Nakaminami et al., 2014) and in Japanese apricot buds following gibberellin treatment (Zhuang et al., 2013). Therefore, the changes in protein might not always be associated with the changes in function.

In garlic, proteome analysis generated 36 proteins whose levels varied significantly between the fertile and sterile flowers; 9 of them presented exclusively in the latter. Within this latter group, a few genes are known to be involved in pollen development. Thus, misregulation of *ADP ribosylation factor* contributes to the defects in tapetal cell division and callose secretion (e.g., in maize, Wang et al., 2012); *SOD* acts as an antioxidant and is involved in sporogenous cell division (e.g., in cotton, Jiang et al., 2007). Our results indicate that at the mid stage of male-sterile flower development, when hypertrophy of the tapetum first becomes evident, the transcript and protein levels of *SOD* are high (Figure 9). It is possible that the stress from ROS production at this developmental stage serves as a signal to increase SOD activity due to a feedback mechanism, as has been described in cotton (Jiang et al., 2007). Later, a very low level of *SOD* expression was registered, when massive pollen abortion occurs (Figures 2, 9). This decrease might result from high ROS level and oxidative damage to the developing pollen.

It is suggested that in garlic, separate transcriptome or proteome analyses are not sufficient to understand the underlying genetic and molecular mechanisms. For a comprehensive interpretation of developmental processes, including mechanisms of male sterility, future research and integration of physiological and anatomical studies with transcriptome, proteome and metabolome analyses are required.

### Future research and practical use of garlic fertility and male sterility

A comparison of morphophysiological and molecular traits of fertile and male-sterile garlic flowers suggests that the most vulnerable stage of pollen development is the post-meiotic, uninuclear stage (Shemesh-Mayer et al., 2015; Figure 2). During this stage, respiratory restrictions caused by a disruption in mitochondrial functions can lead to energy deficiency and consequent pollen abortion. This process can also be linked to oxidative stress, which negatively affects tapetum development and pollen nutrition. Similar to onion, bunching onion and chives, garlic male sterility might result from the interaction between CMS and GMS (CGMS). This notion warrants further investigation. Transcriptome and proteome analyses suggest that garlic homologs of *MS2, MMD1*, and *GPAT2* can be further developed as possible molecular markers for fertility, and *nad7, ccmC, 18S rRNA* as possible markers for male sterility in garlic.

In order to verify CMS gene candidates, functional data analysis is required. One of the main technical barriers to such analysis is the lack of a successful method to transform plant mitochondrial genomes. As an alternative strategy, recombinant constructs of the CMS candidate genes (the chimeric ORFs fused with a mitochondrial targeting signal sequence) from bean, rice, sugar beet, pepper and other plants were produced and transferred into the nuclei of tobacco plants. This procedure caused male sterility in tobacco, thus verifying the CMS functions of the fused genes (Chen and Liu, 2014). The second option can be use of the transformation system in garlic. However, garlic is a bulbous perennial plant with a complicated flower biology, and its transformation system is not available yet. Therefore, unlike in model plants systems, the application of functional data analysis in garlic is not feasible at this point. Future research is needed to define garlic CMS mitochondrial genes and to provide their functional analysis in model plants or in garlic.

Male sterility is known to be a practical and cost-effective mechanism for commercial F1 hybrid seed production in onion (Havey, 2004). Male-sterile plants have a selective advantage: as a result of reallocation of the energy saved from pollen production, they produce more or better seeds than the corresponding hermaphroditic plants (Touzet and Meyer, 2014). Male-sterile garlic clones are characterized by enhanced seed setting compared to fertile plants, and therefore can be used in the commercial production of hybrid seeds. Searching for specific chimeric ORFs fused to mitochondrial genes in garlic will facilitate further identification of molecular markers related to male sterility, as has been done in onion (Kim et al., 2009a), and will enable the future use of male sterility in the commercial hybridization of garlic.

## Author contributions

All authors contributed to the experiment and the manuscript. ESM, AS, AK, and RK designed the experiment and drafted the manuscript. ADF carried out the bioinformatics analysis. ESM, TBM, NR, and DP conducted the experimental work. HDR and AS conceived the study, and contributed to the writing of the manuscript. This research is a part of the graduate studies of ESM and TBM. All authors have read and approved the final manuscript.

### Conflict of interest statement

The authors declare that the research was conducted in the absence of any commercial or financial relationships that could be construed as a potential conflict of interest.
