# Supplementary figures and images for: Garlic (Allium sativum L.) fertility: transcriptome and proteome analyses provide insight into flower and pollen development
Source: Front Plant Sci. 2015 Apr 28;6:271. doi: 10.3389/fpls.2015.00271 (PMC4411974; doi:10.3389/fpls.2015.00271)

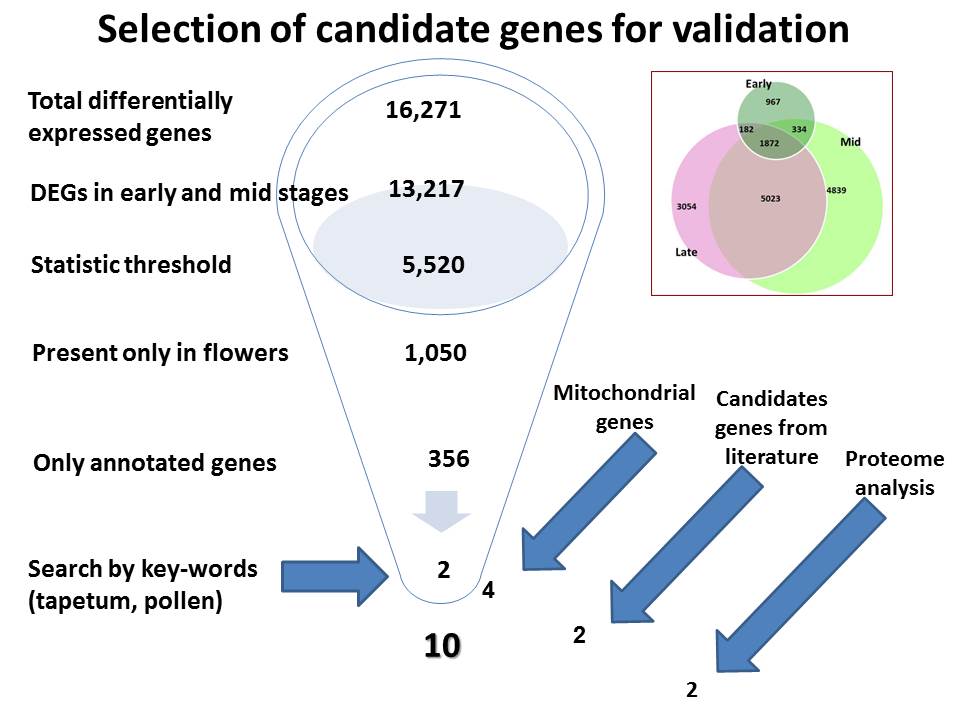

Supplement: Supplementary file 1 [file Image1.JPEG]
